# An Antimicrobial Copper–Plastic Composite Coating: Characterization and In Situ Study in a Hospital Environment

**DOI:** 10.3390/ijms25084471

**Published:** 2024-04-18

**Authors:** Alexandre M. Emelyanenko, Fadi S. Omran, Maria A. Teplonogova, Marina Y. Chernukha, Lusine R. Avetisyan, Eugenia G. Tselikina, Gleb A. Putsman, Sergey K. Zyryanov, Olga I. Butranova, Kirill A. Emelyanenko, Ludmila B. Boinovich

**Affiliations:** 1 A. N. Frumkin Institute of Physical Chemistry and Electrochemistry, Russian Academy of Sciences, Leninsky Prospect 31, 119071 Moscow, Russia; duckyfriedrich@gmail.com (F.S.O.); chernukha08@mail.ru (M.Y.C.); lusavr@mail.ru (L.R.A.); gleb.putsman@gmail.com (G.A.P.); sergey.k.zyryanov@gmail.com (S.K.Z.); butranovaolga@mail.ru (O.I.B.); emelyanenko.kirill@gmail.com (K.A.E.); 2 N. S. Kurnakov Institute of General and Inorganic Chemistry, Leninsky Prospect 31, 119071 Moscow, Russia; ma_teplonogova@igic.ras.ru; 3 Department of Medical Microbiology, Gamaleya National Research Center for Epidemiology and Microbiology, Ministry of Health of the Russian Federation, 18 Gamaleya St., 123098 Moscow, Russia; geshka90@gmail.com; 4 City Clinical Hospital No. 24, Moscow City Health Department, 10 Pistsovaya St., 127015 Moscow, Russia; 5 Department of General and Clinical Pharmacology, Institute of Medicine, Peoples’ Friendship University of Russia named after Patrice Lumumba, 6 Miklukho-Maklaya St. 117198 Moscow, Russia

**Keywords:** bactericidal coatings, healthcare-associated infections, antimicrobial effect, ABS plastic, copper

## Abstract

A method has been proposed for creating an operationally durable copper coating with antimicrobial properties for the buttons of electrical switches based on the gas dynamic spray deposition of copper on acrylonitrile butadiene styrene (ABS) plastic. It is shown that during the coating process, a polymer film is formed on top of the copper layer. Comparative in situ studies of microbial contamination have shown that the copper-coated buttons have a significant antimicrobial effect compared to standard buttons. Analysis of swabs over a 22-week study in a hospital environment showed that the frequency of contamination for a copper-coated button with various microorganisms was 2.7 times lower than that of a control button. The presented results allow us to consider the developed copper coating for plastic switches an effective alternative method in the fight against healthcare-associated infections.

## 1. Introduction

Healthcare-associated infections (HAIs) represent a major problem in modern healthcare, causing serious social and economic harm and having a widespread distribution [1,2]. Among the causes of mortality in the population, HAIs occupy tenth place and affect up to 10% of patients in medical care units [3]. In the Russian Federation, according to the data of the Reference center for monitoring HAIs of the Central Research Institute of Epidemiology, the absolute number of HAI cases was 33,913 in 2021. The real incidence of hospital infections, according to the expert estimates of Russian epidemiologists, is much higher, and the economic burden from HAI cases, according to rough estimates, is about 300 billion rubles (~3 billion US $) per year [3]. The corresponding burden of the US healthcare system was estimated about 10 billion US $ in 2013 [4]. Along with this, the healthcare environment is a reservoir for the cultivation of strains of microorganisms that are resistant to antibiotics and/or disinfectants [5,6,7,8], which further aggravates the multidrug resistance problem. The development of new antibacterial drugs is certainly an extremely important task for global healthcare to combat HAIs, but no less important are precautionary measures that can prevent the spread of pathogens with acquired resistance to antibiotics in hospitals.

The etiological agents of HAIs are bacteria, viruses and fungi that contaminate various surfaces, including elevator buttons, switches, door handles and medical equipment, with which both patients and medical staff come into contact [9]. These microorganisms vary depending on the different departments—surgical units, burn units, intensive care units and maternity wards—where healthcare is provided [10,11,12,13]. The special attention of epidemiologists is focused on the group of ESKAPE pathogens (*Enterococcus faecium*, *Staphylococcus aureus*, *Klebsiella pneumoniae*, *Acinetobacter baumannii*, *Pseudomonas aeruginosa* and *Enterobacter* spp.), which are characterized by an increased level of resistance to several groups of antibiotics [8,14]. Therefore, reducing the risk of contamination of objects in the hospital environment is one of the most important tasks of modern science, especially in terms of preparedness for future pandemic situations [15].

One of the most prospective and demanded directions of research targeted on reducing the dissemination of infections through touch surfaces is the implementation of biomimetic approaches, based on the exploitation of the bactericidal properties of some metals (Ag, Cu, Mg etc.). Metallic components might be incorporated into touch surfaces in the form of immobilized nanoparticles [16], films with a specific roughness [17,18] or nanoparticle-containing composites [19,20]. The antibacterial effect of such surfaces has been repeatedly verified in laboratory studies [21,22,23] but insufficiently assessed under the conditions of clinical practice [24,25,26,27,28,29]. Copper is one of the earliest known biocidal materials [30]. It can inactivate viruses such as coronaviruses [31] and be used against different kinds of bacteria, algae and fungi [32,33].

Objective data characterizing the effectiveness of the use of antibacterial metal coatings in clinical practice can only be obtained on the basis of well-designed controlled studies carried out in healthcare institutions. The crucial points to determine their degree of effectiveness are the qualitative and quantitative characteristics of the microflora from the studied surfaces under the conditions of their routine daily use by patients and medical personnel. The purpose of this study was to develop a simple and economically attractive method for the deposition of a copper coating on the surface of standard commercially distributed plastic light switches and to assess the bactericidal activity of the fabricated coating against HAI pathogens in a hospital setting during regular clinical activity.

## 2. Results and Discussion

### 2.1. Physico-Chemical Properties of the Materials under Study

**Figure 1:**
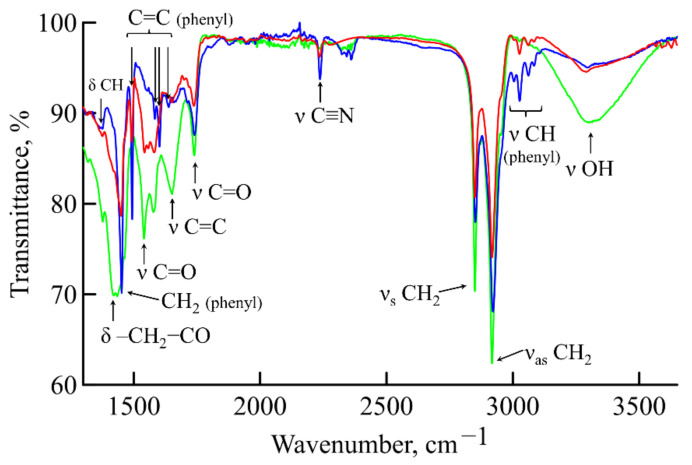
IR spectra from an uncoated ABS plastic switch button (blue), a button with a freshly applied copper coating (red) and a copper-coated button exposed in an intensive care unit for 22 weeks (green).

The buttons of the electrical switches studied in this work were made from ABS plastic, which is a graft copolymer of styrene with acrylonitrile and butadiene. This material is extensively used for the manufacture of various medical products, including electrical products in medical units. To monitor the chemical composition of the buttons used in this work, the IR spectra were obtained and analyzed from an ABS plastic switch button washed in an ultrasonic bath, a button with a freshly applied copper coating and a copper-coated button exposed in an intensive care unit for 22 weeks. The spectrum of the ABS plastic shown in Figure 1 (blue line) corresponds in composition to a copolymer of styrene with acrylonitrile and butadiene. This is indicated by the characteristic bands of the stretching vibrations of the C=C and C–H groups of the phenyl radical, the symmetric and asymmetric stretching vibrations of the CH_2_ groups, the stretching vibrations of the C≡N group and the skeletal vibrations of the aromatic ring [34]. In addition, this spectrum clearly shows the bands of the OH and C=O stretching vibrations, which arise during the thermal oxidation that accompanies the conventional processing of molded electrical products [35,36,37].

It is important to note that on the spectrum of a switch button made of ABS plastic, after applying a layer of copper (red line), the main bands characteristic of the plastic are clearly visible, which indicates the fact that the surface of copper, after its application, is covered with a layer of ABS plastic. Finally, in the spectrum corresponding to a button with a copper coating, which was subjected to contact with the fingers of medical personnel for several months (green line), bands corresponding to new types of vibrations appeared, and at the same time, the intensities of the valence OH and C=O vibrations (1738 cm^−1^) increased. The new bands appearing in the spectrum are consistent with the typical composition of latent fingerprint residues containing amino acids and fatty acids [38].

To explain the observed phenomena of the presence of a polymer film on the surface of the copper coating and the increase in the intensity of the bands characteristic of the acrylonitrile–butadiene–styrene copolymer over its time of operation, let us consider in more detail the structure of the sputtered copper coating and its morphology.

**Figure 2:**
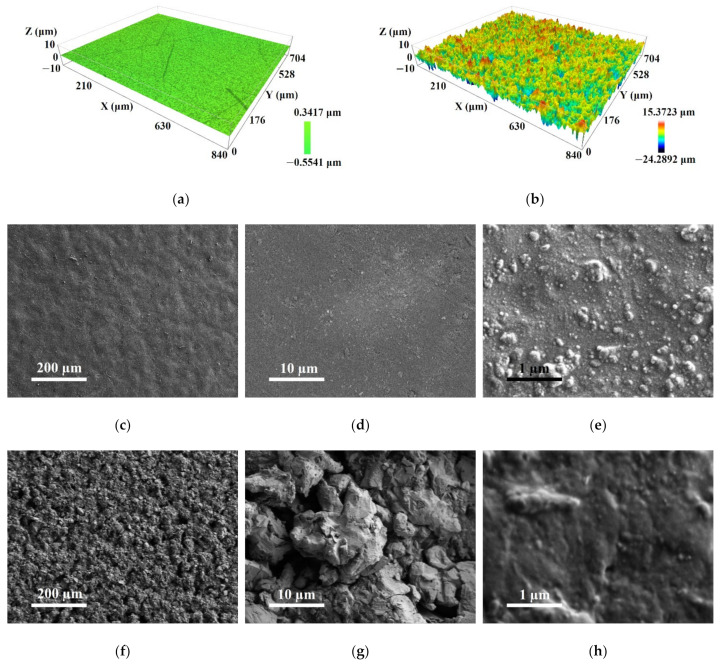
Confocal microscope images (**a**,**b**) and SEM images of the surface of uncoated (**a**,**c**–**e**) and copper-coated (**b**,**f**–**h**) electric switch buttons. SEM images were taken at different magnifications: ×500 (**c**,**f**), ×10,000 (**d**,**g**) and ×100,000 (**e**,**h**).

Figure 2a,b show images of the surfaces of both the coated and uncoated switch buttons obtained using a confocal microscope. Image processing, according to ISO 25178 [39], gives the following roughness parameters for a plastic button: S_ratio_ = 1.0004; S_z_ = 0.90 µm; S_a_ = 0.06 µm. For a button with a sputtered copper coating, the roughness parameters are S_ratio_ = 1.36; S_z_ = 45.7 µm; S_a_ = 2.8 µm. The data obtained indicate the high smoothness of the original plastic button at the micro level and the presence of nanotexture at the nano level. For copper coatings, their roughness is significant at the micro level.

The above observations are well supported, with the SEM images obtained at ×500, ×10,000 and ×100,000 magnifications presented in Figure 2c–h. A distinctive feature of copper coatings is the presence of pores with sizes from 3 to 45 µm (Figure 2f) and a microgranular structure (Figure 2g).

**Figure 3:**
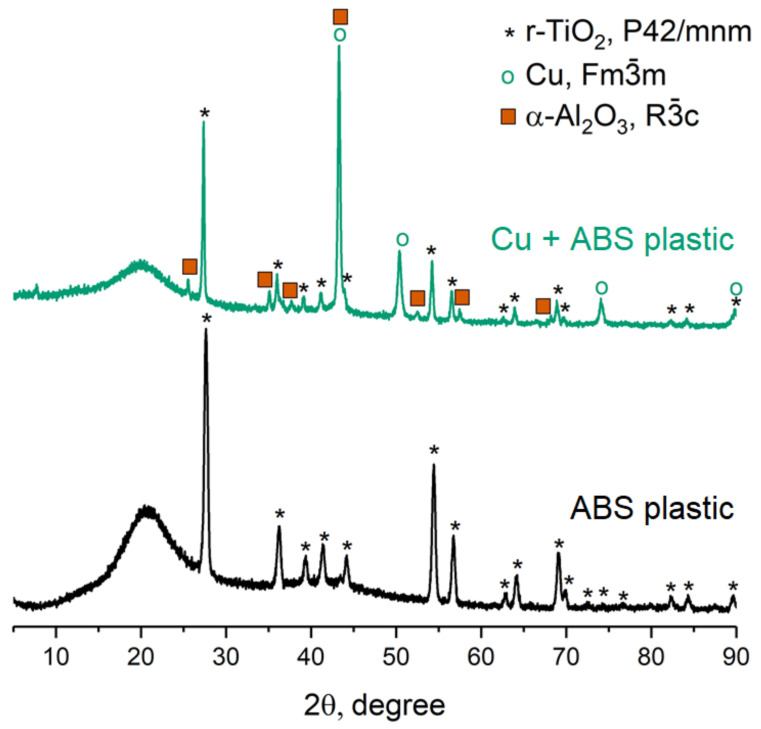
Diffraction patterns of ABS plastic samples before and after copper sputtering on their surface.

The X-ray diffraction patterns of the plastic switch buttons before (ABS plastic) and after (Cu + ABS plastic) the application of a copper coating to their surface are shown in Figure 3. According to our estimations based on the SEM data, the size of the copper particles ranges from 2 to 15 μm (Figure 2g). The halo in both diffraction patterns in the 2θ region of 15–25° indicates that the plastic consists of an X-ray amorphous polymer, e.g., amorphous ABS plastic. Amorphous compounds are known to show such a halo namely in this 2θ region, and the same effect was observed, for instance, in study [40]. The XRD results are consistent with the EDS data analysis, indicating the presence of copper, titanium and aluminum in the ABS plastic switch after the application of a copper coating (Figure S1 in the Supplementary Materials).

Both samples also contain crystalline titanium (IV) oxide TiO_2_ of the rutile modification (space group P42/mnm, PDF2 card no. 21-1276). Titanium oxide is present in the plastic, apparently as an inorganic filler to give the material a light color, and it is clearly visible in Figure 2e in the form of individual nanoparticles. In the diffraction pattern of a sample coated with copper (Cu + ABS plastic), reflections of crystalline copper ($Fm\bar{3}m$, PDF2 no. 4-836) and low-intensity reflections of corundum α-Al_2_O_3_ ($R\bar{3}c$, PDF2 no. 46-1212) appeared. The corundum appeared in the coating because corundum particles are added into the sputtered copper powder as an abrasive for better coating adhesion.

Let us now return to the issue of the appearance of signs of the formation of a polymer film on top of the sprayed metal coating, which manifest in both the IR spectra and the XRD patterns.

Here, it is necessary to emphasize that during the industrial production of ABS plastic, the total degree of comonomer conversion does not exceed 90–95%, i.e., monomers are present in the finished product. When applying a copper coating using the gas dynamic spraying method, there is a significant increase in surface temperature (*T*_max_ < 150 °C), which causes plastic softening and an increase in the rate of diffusion of monomers and oligomers. In this case, oligomers can be formed as a result of the destruction of the polymer under the influence of high temperature and a high-speed beam of copper and corundum particles. As a result of these processes, on the one hand, copper particles are introduced into the softened plastic base, and a composite is formed. On the other hand, polymer chains can break with the formation of low-molecular fragments with a comonomer ratio different from that in the bulk of the plastic. In addition, since the surface energy of the comonomer layer is significantly lower than the surface energy of copper, the transport of monomers along the surface of the pores of the composite during film deposition leads to the formation of a plastic layer on the metal coating. When transporting polymer fragments to the surface, the surface layer will be enriched with a nonpolar component, which has a lower surface energy. Considering that the acrylonitrile and styrene fragments are polar, and the polybutadiene fragment is nonpolar, it is expected that the concentration of polybutadiene in the surface film of the ABS plastic will increase. During product operation, the subsequent diffusion of monomers and their copolymerization causes further thickening of the polymer layer and filling of the pores with the polymer. Thus, the scenario presented here for the formation of a composite copper coating on ABS plastic allows us to explain the obtained IR spectra (Figure 1) and X-ray diffraction patterns (Figure 3) for plastic switch buttons with a copper coating.

**Figure 4:**
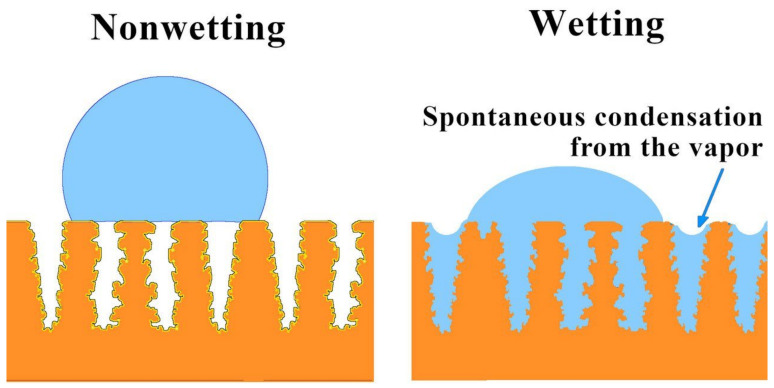
On the nonwetted surfaces (**left**), the droplets of bacterial suspensions take nearly spherical shapes and evaporate faster. In contrast, on the wetted surfaces (**right**), the pores, due to spontaneous water condensation from undersaturated vapors, are filled with water and maintain moisture, thus slowing the evaporation of suspension droplets and promoting biofilm formation.

It has been shown previously that the antibacterial properties of metal surfaces, such as copper, significantly depend on the wetting of these surfaces with aqueous media [17,22,41]. Several factors affect this dependence. Firstly, the spread of bacterial infections in healthcare settings occurs through touch surfaces in the form of aerosols or microdroplets of biological fluids, such as sweat, saliva, urine, etc. [42,43]. All of these fluids contaminated with microorganisms are water-based suspensions. When drops of such suspensions come into contact with a solid surface, the nature of the interaction between, say, the bacterial cells and the surface and the morphology of the biofilms subsequently formed on the surface will largely depend on the wettability of this surface by the bacterial suspension [17,22,44]. Additionally, the intensity of the release of metal ions, which have a toxic effect on bacterial cells, depends on the corrosion processes at the interface between the metal substrate and the bacterial suspension [18]. The intensity of these processes, in turn, is determined by the area of interfacial contact, that is, by the contact angle of the suspension on the substrate [17]. Finally, a good wettability of porous substrates leads to capillary condensation of water vapor from the atmosphere into the pores of the substrate (Figure 4), ultimately resulting in long-term moisture retention on the surface with precipitated bacterial cells. The maintenance of moisture on the porous surface or the slow evaporation of an aqueous dispersion medium promotes cell viability and subsequent biofilm formation [45].

In view of the important role of the wettability of the touch surfaces in understanding their antibacterial effects, in this work, we studied the wetting of both the original plastic switch button and the buttons with a deposited copper coating. In Table 1, we present data on the water contact angles for the original plastic switch and for switches with copper coatings immediately after copper deposition and after long-term exposure in an intensive care unit. To measure the contact angles, we used the sessile droplet method, which allowed us to measure not only the contact angles but also the surface tension of a water droplet placed on the test surface. Knowing the surface tension is very important for analyzing the effects of surface contaminants on the contact angle. From the data presented in Table 1, it can be seen that the surface tension of the water droplets on the surface of ABS plastic and the buttons with a freshly applied copper coating corresponds to that of deionized water. This indicates that there are no surfactants present on these surfaces that could readily dissolve in water and reduce its surface tension. In contrast, the surface tension of the water droplet on a copper-coated switch button that has been exposed for a long time in a hospital environment reduces significantly within a few seconds. This indicates a transfer of soluble surfactant impurities from the coating/droplet interface to the droplet/air interface. These results are in good agreement with the infrared spectroscopy data showing the presence of amino acids and fatty acids on the button’s surface after contact with personnel’s fingers (Figure 1, green line).

As follows from the table, the contact angle of ABS plastic with water is 86°, which is slightly less than 90°. This classifies ABS plastic as a slightly hydrophilic polymer. For comparison, the contact angle of polystyrene acrylonitrile is 78° according to [46], further supporting the idea that the non-polar moiety of the polybutadiene chain contributes to the more hydrophobic properties of ABS plastic. Since the contact angle of the switch button immediately after copper deposition approached 150°, two conclusions can be drawn. Firstly, the destruction of the upper polymer layers during the copper deposition causes the concentration of polybutadiene in the surface layers of the ABS plastic to be significantly higher than in the bulk, resulting in the achievement of hydrophobic properties in the coating. Secondly, the very high contact angle can be attributed to the influence of roughness on the effective contact angle. And finally, the high wetting hysteresis of the copper-coated surface, at which a 15 μL droplet of water remains held on a vertically oriented plastic surface, indicates the establishment of a highly hydrophobic surface state in the homogeneous (Wenzel) wetting mode. The fact that the contact angle of the switch button after the clinical studies was much lower is in good agreement with the above-mentioned accumulation of amino acids on the surface of the button during repeated contact with the fingers of personnel.

### 2.2. Study of Bactericidal Properties

A recent extensive review [47] examining the potential of various forms of copper-treated surfaces in hospital rooms to reduce the risk of HAIs concluded that the 7 studies reviewed showed a 27% reduction in HAIs (risk ratio 0.73, 95% confidence interval 0.57–0.94; I^2^ = 44%, *p* = 0.01). The studies reviewed were performed using copper on surfaces such as bed rails, door handles, table tops and patient bed linens, gowns and towels. At the same time, such hard touch surfaces as the buttons of electrical switches, without the use of which the functioning of a modern medical institution is impossible, were not included. To fill this gap, in this study, we focused specifically on analyzing the reduction in the contamination of such hard surfaces as electrical switch buttons.

The peculiarities of these objects are that modern materials science can offer a wide range of different methods for applying copper to such switches in order to select the most effective coating, both in terms of its bactericidal properties and taking into account economic feasibility, aesthetic properties and durability. Since the buttons of electrical switches are currently mainly made of plastic, we used ABS plastic, which occupies a large share of the electrical products market, as the base for the coating. The copper coatings applied to such buttons showed high mechanical resistance and durability throughout the entire period of clinical studies, which was the basis for the long-term preservation of the bactericidal properties we discovered. A comparative analysis of the contamination of a plastic button and a copper-coated button was carried out based on two different approaches. One of the approaches was based on a binary assessment of the contamination of the test surface, when in the presence of the growth of at least one type of microorganisms, either on a solid nutrient medium or in an accumulation medium, the result of the study was assigned a value of 1. If, when analyzing the swab, no growth of microorganisms was detected, then the study result was assigned a value of 0.

**Figure 5:**
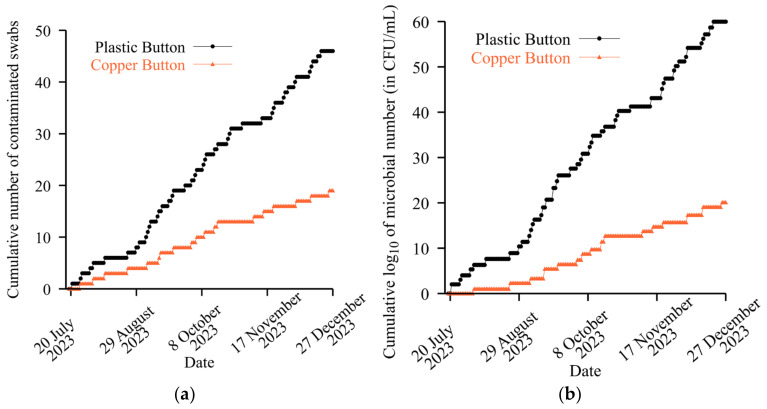
The evolution of total number of swabs in which microorganisms were found (**a**) and of the sum of logarithms of the total microbial numbers (**b**) with the time of clinical trials.

The results on the contamination of both buttons obtained as a result of this approach are presented in Figure 5a, where the abscissa axis shows the date of swab collection and the ordinate axis shows the cumulative total number of swabs in which microorganisms were found. To take into account the level of contamination using such a binary approach, the swab analysis result can be assigned not a value of 1 when microorganisms are detected but a value equal to the decimal logarithm of the total microbial number (Figure 5b). In this case, the ordinate axis shows the (cumulative) sum of the logarithms of the total microbial numbers at a specific moment of observation.

The bactericidal properties of the tested switches were judged by the rate of change in the total number of cases of detection of microorganisms depending on the time of the clinical trials. Both types of binary data analysis presented in Figure 5 unambiguously indicate a notable reduction in microbial contamination on the surface of the copper coating with respect to the ABS plastic button.

The maximum and average levels of microbial contamination for the entire period of clinical trials are presented in Table 2. The data obtained show that out of 89 swabs from the plastic switch button, microorganisms were detected in 56 cases (48 cases on solid nutrient media and 8 in accumulation media). At the same time, for the switch button with a copper coating, only in 21 cases out of 89 was the growth of microorganisms observed, of which 16 cases were on solid nutrient media and 5 cases were in accumulation media. Thus, the frequency of the contamination of the switches with a copper coating, with a significance level of *p* < 0.05, was 63% lower than the contamination of standard plastic switches. At the same time, the maximum contamination of the plastic switch button during the entire observation period was 400 colony-forming units per mL (CFU/mL), with an average level of 17.8 CFU/mL. For the copper-coated switch button, the observed maximum microbial contamination level was 130 CFU/mL, with an average contamination level of 4.8 CFU/mL. Thus, with a significance level of *p* < 0.05, during the entire period of clinical trials, the maximum contamination on the switch button with a copper coating was 3 times lower, and the average level of contamination was 3.7 times lower than for a plastic switch button.

It seemed interesting to compare the frequency of the contamination of the test samples with various representatives of the human microbiome and the environment. Table 3 shows the number of cases of detection of various microorganisms, and the relative frequencies of detecting each type of contaminant in the total microbial contamination. It should be noted here that since several types of microorganisms were simultaneously detected in a number of swabs from a plastic switch button, the total number of detections of all types of microorganisms (64) was higher than the number of contaminated swabs (56, see Table 2). For the switch button with a copper coating, the case of simultaneous contamination by different microorganisms occurred only once.

Thus, over 22 weeks of clinical observations, the only detected ESKAPE pathogen (*Staphylococcus aureus*) was found 8 times on the plastic switch button and 3 times on the copper-coated switch button. The main type of pathogenic bacterial contamination of the buttons was *Staphylococcus epidermidis*, which was found (with *p* < 0.05) 2.7 times less often on the button with a sputtered copper coating than on the control plastic button.

## 3. Materials and Methods

### 3.1. Objectives and General Design of the Study

The objects of the study were commercial electrical switches based on acrylonitrile butadiene styrene plastic (ABS plastic), widely used in medical institutions. The appearance of the switch is shown in Figure S2 in the Supplementary Materials. Comparative studies to assess the bacterial contamination were carried out using three buttons, one of which was made of common ABS plastic (this button is white in the photo), while the other two were obtained by applying a bactericidal copper coating to the ABS plastic (red-brown color). The contact surface area of the plastic button was equal to the sum of the surface areas of the two coated buttons and amounted to 24.5 cm^2^. The buttons were installed in the intensive care unit of Moscow City Clinical Hospital. According to the standard protocol [48], all touch surfaces in the department are wiped twice a day with Avansept Active disinfectant, based on alkylamine and polyhexamethylene biguanide. When selecting the research objects, it was taken into account that the department staff consisted of 20 people, and the frequency of the medical personnel pressing a plastic button and simultaneously pressing two coated buttons coincided and amounted to up to 100 times a day. To assess the contamination of the buttons with various microorganisms, swabs were taken from the buttons before the first daily wipe with a disinfectant 4 times a week for about 22 weeks.

### 3.2. Materials

The copper powder for deposition on the surface of the plastic buttons was obtained from Obninsk Center for Powder Spraying (OCPS, Obninsk, Russia). Sterile cotton swabs for taking the swab samples were purchased from LLC MiniMed (Bryansk, Russia). The meat-peptone broth (MPB), mannitol salt agar with the addition of egg yolk, Sabouraud agar and Endo agar were obtained from the State Research Center for Applied Biotechnology and Microbiology (Obolensk, Russia). To cultivate the bacteria, we used blood agar prepared from defibrillated cattle blood serum (LeiTran LLC, Moscow, Russia) and Nutrient medium No. 1 (Obolensk, Russia). Type I ASTM deionized water was used to hydrate the swab tampons, while sterile physiological solution (0.9 wt.% NaCl, PanEco, Moscow, Russia) served as a transport medium for the swabs.

### 3.3. Preparation of the Copper Coatings

To apply a copper layer on the ABS plastic button surface, we used gas dynamic spray technology (also known as “Low-Pressure Cold Spraying”) [49]. The spraying equipment DYMET^®^ model 423 (OCPS, Obninsk, Russia) was used to spray the copper powder over the plastic surface. The copper coating was deposited layer by layer until it reached a thickness of approximately 40 µm. An average of 5 layers were required to obtain this thickness. Since the application method involves the dynamic impact of the metal micro- and nanoparticles on the surface, when applying a coating to plastic, the plastic base heats up and softens. In addition, as shown in the Section 2, heating the plastic leads to accelerated diffusion of monomers and oligomers onto the surface of the sprayed coating, additional binding of the metal particles in the coating and a decrease in the surface energy of the obtained composite surface. Therefore, deposition was performed by moving the nozzle of the air gun over the surface of the switch at a speed of about 5 mm/s and at a distance of about 10–20 mm, with a 10 s pause after each pass to allow the plastic to cool.

### 3.4. Characterization of the Coatings

The structure, morphology and surface chemistry of the tested electrical switches were examined using confocal microscopy, scanning electron microscopy, energy-dispersive X-ray spectroscopy, X-ray diffraction and IR spectroscopy.

Electron microscopic images of the surface were obtained using a TESCAN AMBER GMH microscope (TESCAN, Brno, Czech Republic) at an accelerating voltage of 1 keV using an Everhart–Thornley secondary electron detector.

Energy-dispersive X-ray spectroscopy was carried out at accelerating voltages of 20 kV using an Oxford Instruments Ultim MAX EDS detector calibrated against a cobalt standard. The EDS data were processed using AZtec 5.0 SP1 software. Before the analysis, the samples were coated with a carbon layer.

X-ray diffraction analysis (XRD) of the samples was carried out using a Haoyuan DX-2700BH diffractometer (Haoyuan Instrument, Dandong, China) using CuK_α_ radiation (λ = 1.54184 Å) in the 2θ range of 5–90° with a step of 0.02° and a shutter speed of 0.5 s/step. The Bragg–Brentano geometry was applied. The samples were fixed at a cuvette position and aligned.

The FT-IR spectra of the powders were taken in the range of 400–4000 cm^−1^ using an InfraLUM FT-08 (Lumex, St. Petersburg, Russia) device in the attenuated total reflectance mode with a diamond crystal. The spectra accumulation time was 1 min (93 scans), and the spectral resolution was 4 cm^−1^.

To obtain the information on the surface relief and roughness of the deposited copper coating, we used the 3D optical profilometer S neox (Sensofar Metrology, Barcelona, Spain). The SensoSCAN 2.0 software facilitated advanced analysis and visualization of the surface microtopography.

Since the wettability of a touch surface is an important factor significantly affecting the antibacterial efficiency of the surface, the wettability parameters of the tested coatings in this study were determined both before and after completing the clinical experiment. The contact angle and the surface tension of a drop of deionized water placed on the test surface were determined from the image of a sessile drop, formed using a setup described in detail in [50]. To determine the wettability parameters, digital processing of the image of a sessile drop was used, followed by a search for the parameters of the Laplace curve describing the experimental profile of the drop in an optimal way. In addition, for a more detailed characterization of the surface wetting, the sliding angle of a drop of water with a volume of 15 µL from the test surface was also determined. Both the contact and sliding angles reported in this work were determined by averaging over the values obtained at 10 different points on the surface under study.

### 3.5. Collection of Swabs

When collecting swabs from the surfaces, sterile cotton swabs were used. The swab was moistened with deionized water immediately before collection. Swabs were taken from the entire surface of the switch buttons, with one swab from a plastic button and the second from both copper-coated buttons. After collecting the probe, each swab was placed in an individual transport container containing 2 mL of 0.9% NaCl, with the accompanying information (sampling point and date) pre-applied. The delivery time to the laboratory where the inoculation on nutrient media was carried out did not exceed 6 h from the moment the probe was taken.

### 3.6. Assessment of the Test Surface Contamination and Identification of the Contaminants

For the cultivation of the microorganisms collected from the test surfaces, 0.1 mL of liquid from each transport container was transferred into a test tube, with 2 mL of MPB serving as an accumulation medium. Simultaneously, several other portions of 0.1 mL each were seeded on Petri dishes with dense nutrient media to identify particular possible pathogens able to cause HAIs. To reveal *Staphylococcus aureus*, mannitol salt agar with the addition of egg yolk was used. To isolate enterobacteria, seeding was performed on either Endo agar or the blood agar prepared from defibrillated cattle blood serum and Pronadisa agar. Sabouraud agar was used for the identification of fungi. The Petri dishes with Sabouraud agar were incubated at 30 °C for 48–72 h and those with mannitol salt agar at 37 °C for 48 h. The inoculated tubes with MPB and the Petri dishes with other solid nutrient media were incubated at 37 °C for 24 h. After the indicated time of incubation for the Petri dishes, the grown colonies were counted, and a pure culture was collected for mass spectroscopic identification. As regards the inoculated tubes with MPB (accumulation medium), the medium from the test tubes for which the turbidity was observed was seeded on the Petri dishes with blood agar for further incubation at 37 °C for 24 h, followed by counting the grown colonies and culture identification as described above.

Microorganism identification was performed using Matrix-Assisted Laser Desorption/Ionization Time-Of-Flight Mass Spectrometry (MALDI–TOFMS) technology on Bruker’s commercial MALDI TOF MS systems ultrafleXtreme and Biotyper (Bruker Daltonics GmbH, Bremen, Germany), using flexControl version 3.4 software with MBT Compass version 4.1 software (Bruker Daltonics). Each spectrum was interpreted using MALDI Biotyper (version 4.0) automation control and the Bruker Biotyper 4.1 software and library (Bruker Daltonics).

### 3.7. Statistical Analysis

Statistical analysis of the obtained data was carried out using Statistica 10 software (StatSoft, Moscow, Russia). To assess the presence of differences in the quantitative and qualitative characteristics in the two groups, the Mann–Whitney U test and Pearson’s chi-square criterion were used, accordingly, at a significance level of *p* ≤ 0.05.

## 4. Conclusions

In this work, a simple and economically attractive method was proposed for creating an operationally durable sputtered copper coating for buttons of electrical switches that exhibits bactericidal properties. The structure of the coating, its composition and its surface morphology were studied. It was shown that during the sputtering process, a polymer film is formed on top of the copper layer. This coating structure is important to protect personnel and patients from direct contact with copper particles, which, in some cases, can cause a number of negative consequences, associated, for example, with skin reactions [27,51]. The influence of repeated contact of the personnel’s fingers with the coating was investigated, and it was shown that although some change in the wetting of the coating occurs, there is no significant change in the composition and morphology of the surface layer of the coating and its bactericidal properties.

Comparative studies of the contamination of an ABS plastic button and a copper-coated button of an electric switch showed that the latter had a significant antimicrobial effect. Analysis of swabs from the plastic and the copper-coated buttons over a period of 22 weeks showed that the frequency of contamination for the copper-coated button with various microorganisms was 2.7 times lower than for the control button. At the same time, the main type of pathogenic bacterial contamination of the electric switch buttons was *Staphylococcus epidermidis*. The frequency of contamination with this particular microorganism on the copper-coated button was 2.7 times lower than on the control plastic button (with a significance level of *p* < 0.05).

In general, the results presented here allow us to consider the developed copper coating for plastic switches as an effective alternative method in the fight against healthcare-associated infections, and the coating itself, which exhibits durable physicochemical and antibacterial properties, is promising for practical use in healthcare facilities.

## Figures and Tables

**Table 1:** Contact angle, surface tension and images of water droplets on tested touch surfaces.

Sample	Contact Angle, °	Surface Tension, mN/m	Droplet Image
Initial ABS plastic switch button	85.6 ± 1.7	72.1 ± 0.3	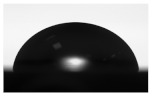
Plastic switch button with freshly sputtered copper coating	149.8 ± 2.6	72.2 ± 1.4	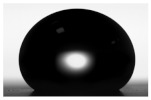
Plastic switch button with copper coating after exposure in hospital environment	104.3 ± 4.0	66.5 ± 2.6	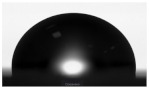

**Table 2:** The contamination levels of the plastic switch button and the copper-coated switch button.

Sample	Total Swabs	Growth Cases		Maximum Total Microbial Number (CFU/mL)	Average Total Microbial Number (CFU/mL)
		Absolute	Fraction (%)		
ABS plastic switch button	89	56	63	149.8 ± 2.6	72.2 ± 1.4
Copper-coated switch button	89	21	24	104.3 ± 4.0	66.5 ± 2.6

**Table 3:** Species and genus of microorganisms isolated from test switch buttons.

Microorganisms	Plastic Switch Button		Copper Coated Switch Button	
	Number of Cases	Fraction, %	Number of Cases	Fraction, %
*Staphylococcus epidermidis*	38	59.4	14	63.6
*Staphylococcus aureus*	8	12.5	3	13.6
*Staphylococcus hominis*	1	1.6		0.0
*Corynebacterium* spp.	3	4.7		0.0
*Micrococcus luteus*	6	9.4	1	4.5
*Bacillus* spp.	4	6.3	2	9.1
*Filamentous fungi*	1	1.6		0.0
*Actinomyces oris*		0.0	1	4.5
*Psychrobacillus psychrotolerans*		0.0	1	4.5
*Aspergillus* spp.	2	3.1		0.0
*Candida Krusei*	1	1.6		0.0
Total	64		22	

## Data Availability

All the data are contained within the article.

## Supplementary Materials

The following supporting information can be downloaded at https://www.mdpi.com/article/10.3390/ijms25084471/s1.
