# Supplementary material for: An Antimicrobial Copper–Plastic Composite Coating: Characterization and In Situ Study in a Hospital Environment
Source: Int J Mol Sci. 2024 Apr 18;25(8):4471. doi: 10.3390/ijms25084471 (PMC11050275; doi:10.3390/ijms25084471)
Supplement: Supplementary file 1 [file ijms-25-04471-s001.zip › ijms-2957169-supplementary.pdf]

Supplementary Information

An Antimicrobial Copper–Plastic Composite Coating: Characterization and In Situ Study in a Hospital Environment

Alexandre M. Emelyanenko [1,*], Fadi S. Omran [1], Maria A. Teplonogova [2], Marina Y. Chernukha [1,3], Lusine R. Avetisyan [1,3], Eugenia G. Tselikina [3], Gleb A. Putsman [1,4], Sergey K. Zyryanov [1,5], Olga I. Butranova [1,5], Kirill A. Emelyanenko [1], Ludmila B. Boinovich [1,*]

[1] A. N. Frumkin Institute of Physical Chemistry and Electrochemistry, Russian Academy of Sciences, Leninsky Prospect 31, 119071 Moscow, Russia
[2] N. S. Kurnakov Institute of General and Inorganic Chemistry, Leninsky Prospect 31, 119071 Moscow, Russia;
[3] Department of Medical Microbiology, Gamaleya National Research Center for Epidemiology and Microbiology, Ministry of Health of the Russian Federation, 18 Gamaleya St., 123098 Moscow, Russia
[4] City Clinical Hospital No. 24, Moscow City Health Department, 10 Pistsovaya St., 127015 Moscow, Russia
[5] Department of General and Clinical Pharmacology, Institute of Medicine, Peoples' Friendship University of Russia named after Patrice Lumumba, 6 Miklukho-Maklaya St. 117198 Moscow, Russia
* Correspondence: ame@phyche.ac.ru (A.M.E.); boinovich@mail.ru (L.B.B.); Tel.: +7-495-955-4625 (A.M.E.)

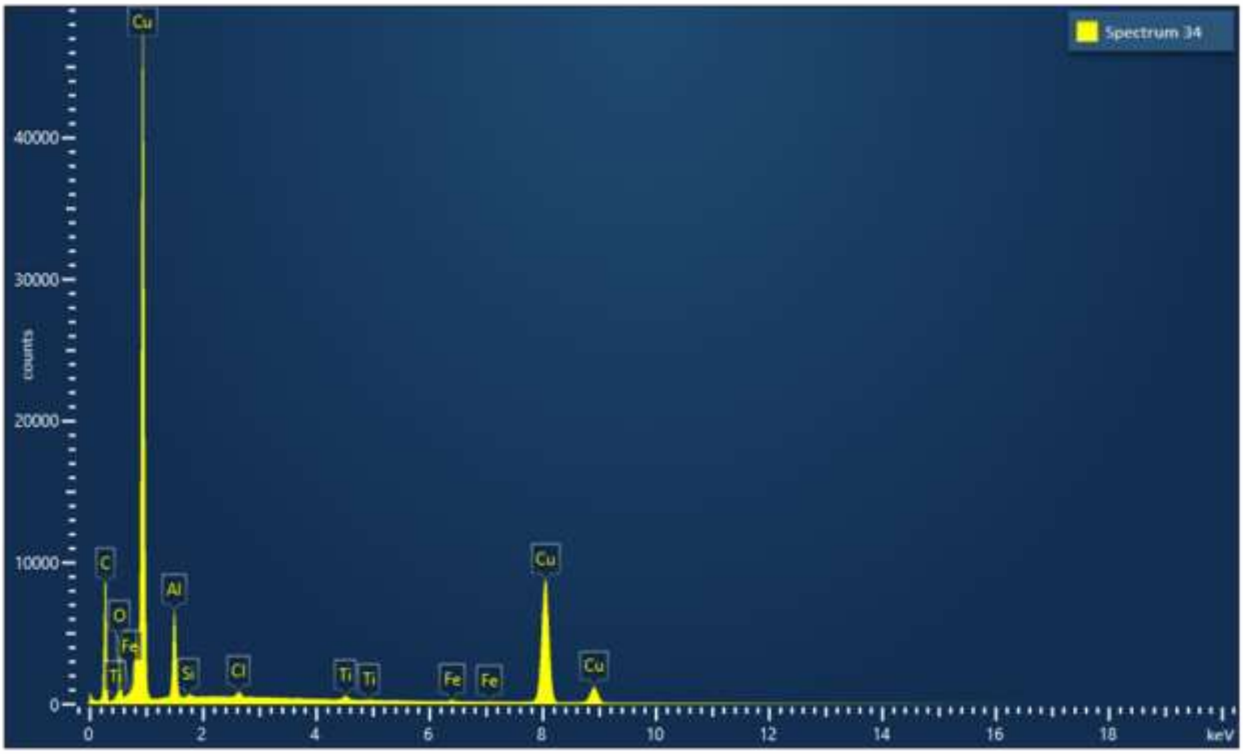

Figure S1. Typical EDS spectra of the ABS plastic switch after application of a copper coating.

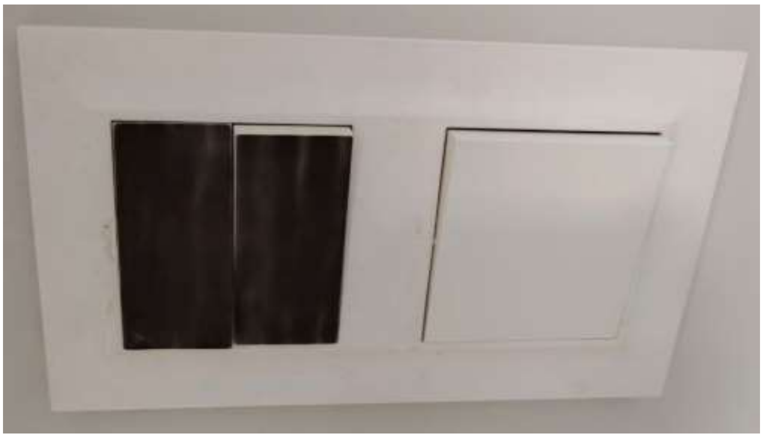

Figure S2. Electric light switch: left paired buttons were coated with a copper layer whereas the right single button was uncoated common ABS plastic.
